# Nanocomposite fibers based on cellulose acetate loaded with fullerene for cancer therapy: preparation, characterization and in-vitro evaluation

**DOI:** 10.1038/s41598-023-48302-2

**Published:** 2023-11-29

**Authors:** Gomaa El Fawal, Marwa M. Abu-Serie, Safaa M. Ali, Noha A. Elessawy

**Affiliations:** 1 Polymer Materials Research Department, SRTA-City), Advanced Technology and New Materials Research Institute, City of Scientific Research and Technological Applications, New Borg El-Arab City, Alexandria 21934 Egypt; 2 Medical Biotechnology Department, Genetic Engineering and Biotechnology Research Institute, City of Scientific Research and Technological Applications (SRTA-City), New Borg EL-Arab City, Alexandria, 21934 Egypt; 3 Nucleic Acid Research Department, Genetic Engineering and Biotechnology Research Institute (GEBRI), City of Scientific Research and Technological Applications (SRTA-City), New Borg El-Arab City, Alexandria 21934 Egypt; 4 Computer Based Engineering Applications Department, Informatics Research Institute IRI, City of Scientific Research and Technological Applications (SRTA-City), New Borg El-Arab City, Alexandria 21934 Egypt

**Keywords:** Cancer, Genetics, Materials science, Nanoscience and technology

## Abstract

The current prevalence of cancerous diseases necessitates the exploration of materials that can effectively treat these conditions while minimizing the occurrence of adverse side effects. This study aims to identify materials with the potential to inhibit the metastasis of cancerous diseases within the human body while concurrently serving as therapeutic agents for their treatment. A novel approach was employed to enhance the anti-cancer properties of electrospun cellulose fibers by incorporating fullerene nanoparticles (NPs) into cellulose acetate (CA) fibers, resulting in a composite material called Fullerene@CA. This development aimed at utilizing the anti-cancer properties of fullerenes for potential therapeutic applications. This process has been demonstrated in vitro against various types of cancer, and it was found that Fullerene@CA nanocomposite fibers displayed robust anticancer activity. Cancer cells (Caco-2, MDA-MB 231, and HepG-2 cells) were inhibited by 0.3 and 0.5 mg.g^−1^ fullerene doses by 58.62–62.87%, 47.86–56.43%, and 48.60–57.73%, respectively. The tested cancer cells shrink and lose their spindle shape due to morphological changes. The investigation of the prepared nanocomposite reveals its impact on various genes, such as BCL2, NF-KB, p53, Bax, and p21, highlighting the therapeutic compounds' effectiveness. The experimental results demonstrated that the incorporation of NPs into CA fibers resulted in a significant improvement in their anti-cancer efficacy. Therefore, it is suggested that these modified fibers could be utilized as a novel therapeutic approach for the treatment and prevention of cancer metastasis.

## Introduction

On a global scale, cardiovascular disease stands as the leading cause of mortality. However, the treatment of cancer is progressively emerging as a formidable challenge due to its intricate nature and limited efficacy of pharmaceutical interventions^1,2^. It is common for most types of malignancies to have intra-tumor heterogeneity, which is connected to disease progression and inadequate therapy response^3^. The available treatment modalities for metastatic cancer include medication, surgery, and radiation; however, chemotherapy has proven to be the most effective and efficient method in clinical practice^4,5^. Most cancer drugs work by inhibiting cancer cell growth, division, and reproduction chemotherapy. However, it has some disadvantages, including severe systemic side effects, toxicity, resistance, and limited selectivity^6^. The medicinal inorganic chemistry field can potentially contribute to the development of innovative pharmaceuticals. One notable example is the clinical utilization of carbonaceous nanomaterials^7^.

Due to their significant advantages over their free drug equivalents, nanoparticles (NPs)-based drug delivery systems (NDDSs) are becoming increasingly attractive for cancer therapy^8–10^. The enhanced permeability and retention (EPR) effect is a widely recognized phenomenon that allows NPs ranging from 10 to 100 nm in diameter to selectively penetrate and accumulate in solid tumors. This is due to the hyperpermeability of vascular walls and the limited drainage of lymphatic drainage in these tumors^11,12^. Several alternative routes have been developed to circumvent the problems associated with standard chemotherapy^13–15^.

Graphene-based nanomaterials with distinctive inherent features include graphene oxide (GO), fullerenes, carbon nanotubes (CNTs), nanodiamonds, and graphene quantum dots (GQDs). The physicochemical properties of this material, encompassing thermal, optical, electrical, mechanical, and structural attributes, confer upon it a distinct advantage over alternative nanoparticles. These properties endow it with enhanced versatility, durability, and electrical conductance in relation to biological entities, thereby facilitating its utility in medical diagnosis and treatment^16,17^. Fullerene, a carbonaceous nanomaterial with significant penetration into solid tumors and strong chemical reactivity, has received substantial attention in cancer theranostics^18,19^. In addition, it is possible to functionalize fullerenes to give them unique physicochemical properties including biocompatibility and water solubility^20,21^. Moreover, a combined application of the fullerene nanocore's photodynamic action can be employed to increase the effectiveness of chemotherapy^20^. It is worth mentioning that the anti-cancer activity of fullerene, along with its sensitization effect on cancer cells, renders it a potent antineoplastic agent. Moreover, it can be used also as an antioxidant and a free radical scavenger^22^. Consequently, numerous researchers used fullerenes as an efficient targeted anti-cancer delivery system. For instance, prostate cancer cells (PC3 cells) were targeted using Docetaxel-loaded polyethyleneimine fullerene with a folic acid-passivated surface^23^. In contrast, doxorubicin (DOX) conjugation with C60 fullerene was reported as an effective treatment against different kinds of cell lines such as Human colon adenocarcinoma cell line (HCT116) and Human Breast cancer cell line (MCF7)^24^. Furthermore, a cationic fullerene based siRNA nanocomplex was used for effective inhalation of T&P&siPD-L1 which could inhibit the growth of metastatic lung cancer without apparent adverse effects and toxicity^25^.

Nanofibers are frequently utilized as carriers for loading bioactive compounds, particularly those that exhibit low water solubility^26,27^. The development of nanofibers represents a remarkable breakthrough in the field of nanotechnology, particularly in enhancing the efficacy of the mass transport of nanotechnology^28^. The advantages of cellulose acetate (CA) over other polymeric fibers are numerous. These advantages include biocompatibility, insolubility in water, biodegradability, exceptional mechanical properties, relatively low manufacturing costs, high affinity, and excellent chemical resistance. CA is particularly suitable for usage in drug delivery systems due to its unique hydrophilicity properties^24^. Several applications of CA have been reported in the literature, including affinity membranes, antimicrobial membranes, biomedical nanocomposites, filament-forming matrices, and biomedical separation^28–30^.

Pharmacological combination approaches have garnered significant attention in order to enhance treatment efficacy and mitigate the occurrence of side effects^31,32^. Combination chemotherapy refers to the concurrent administration of multiple drugs that have distinct mechanisms of action and side effect profiles in order to address multidrug resistance. Nevertheless, the implementation of combination therapy can effectively mitigate the adverse effects associated with single-drug therapy by targeting multiple signaling pathways^33,34^. Clinical practice has also shown synergistic benefits higher than the sum of individual medication effects, as well as decreased systemic toxicity associated with administering lower drug dosages^35^.

Furthermore, encapsulation serves as an effective strategy for reducing the toxicity of materials while preserving their functionality, rendering it a viable technique in the field of biomedical applications^36^. The containment of toxic substances within a shielding enclosure can substantially mitigate the potential deleterious effects on living organisms. This holds significant importance within the realm of biomedicine, wherein the paramount considerations revolve around the safety and efficacy of materials. The process of encapsulation serves to effectively isolate toxic components, preventing their interaction with adjacent tissues or organs, thereby reducing the potential for minimizing any adverse effects^37^. Additionally, the encapsulated materials retain their activity, allowing for their intended use in biomedical applications. Whether it is drug delivery systems or implantable medical devices, encapsulation offers a safe and efficient means of utilizing potentially toxic materials in the field of biomedicine, thereby advancing the progress of medical research and improving patient care. Fullerene, a toxic substance with limited clinical applications, is being investigated for encapsulation in order to mitigate its toxicity. This study represents a novel approach in the field of biomedical applications, as it is the first instance of successfully encapsulating fullerene with a safe dosage through the utilization of electrospinning.

A cyclin protein called Cyclin D regulates the progression of a cell's cycle. The creation of cyclin D, which starts during the G1 phase, drives the transition from G1 to the S phase^38,39^. Cyclin D is one of the most significant cyclins ever produced in terms of functional importance. Four Cdks -Cdk2, 4, 5, and 6- are involved in its interactions^40,41^. In proliferating cells, the accumulation of the cyclin D-Cdk4/6 complex is essential for the progression of the cell cycle. The D-Cdk4/6 cyclin complex partially phosphorylates retinoblastoma tumor suppressor protein, and its inactivation may result in the expression of numerous genes necessary for S phase progression^42^.

This research aims to develop a potential anticancer treatment that targets cancer stem cells while causing less damage in normal cells by using novel nanocomposite electrospun fiber consisting of fullerene NPs prepared from thermal catalytic cracking of mineral water waste bottles then loaded into CA nanofiber and its selective toxicity was tested in vitro using different cancer cell (Caco-2, MDA-MB 231 and HepG-2 cells).

## Materials and methods

Thermo Fisher Scientific provided CA (average Mw = 4 kDa, 39.8 wt percent acetyl) (Waltham, Massachusetts, USA), dichloromethane, and acetic acid from (Sigma in St. Louis, Missouri). Dimethyl sulfoxide (DMSO) was purchased from Merck (Germany). Dulbecco's Modified Eagle's Medium (DMEM) media were purchased from Lonza (USA), while fetal bovine serum (FBS) was purchased from GIBCO Company (USA). In addition, 3-(4, 5-dimethylthiazol-2-yl)-2, 5-diphenyltetrazolium bromide (MTT) was purchased from Sigma Aldrich (Germany). SYBR-green PCR assay kit, cDNA synthesis kit, and Gene JET RNA purification kit were purchased from Thermo Fisher Scientific (Waltham, Massachusetts, USA). Without additional purification, all compounds were of analytical quality and were utilized.

### Preparation of spinning solutions

Many experiments with dichloromethane and acetic acid were carried out to determine the optimal concentration of acetic acid to produce bead-free fibers. Finally, CA (10%) in acetic acid: dichloromethane (1:1) might produce smooth and bead-free fibers. Subsequently, Fullerene@CA fiber was made using different concentrations of fullerene (0.1, 0.3, and 0.5) mg produced by thermal catalytic cracking of mineral water waste bottles^43,44^ to one gram of polymer.

### Fabrication of fullerene@CA nanocomposite fibers

The nanocomposite fibers were prepared using an electrospinning apparatus consisting of a flow rate machine, a metal plate (used as a collector), and a high voltage source. Fibers were gathered (coated with aluminum foil) using a collector. The experimental conditions encompassed a spinning flow rate of 3 ml/h, a spinning voltage of 17 kV, a receiving collector distance of 20 cm, and the experiments were conducted at room temperature. Following the completion of the electrospinning process, the electrospun Fullerene@CA nanocomposite fiber was subjected to a vacuum oven at ambient temperature for a duration of 24 h in order to remove any residual solvent.

### Characterization of fullerene@CA nanocomposite fibers

The surface morphology of all the electrospun Fullerene@CA nanocomposite fibers was observed using Transmission Electron Microscope (TEM, JEOLJEM 1230, Japan) and Scanning Electron Microscopy (SEM, JEOL JSM, Japan) after coating with gold. The softwarImage-Pro 6.0 image analysis software was used to measure the diameter of each sample, and the average value was calculated from randomly selected about 60 measurements. FTIR spectra were captured using the Shimadzu FTIR-8400 S (Japan) instrument from 4000 to 400 cm^−1^ wavelength with a 4 cm^−1^ resolution for all spectra.

### Determination of fullerene release profile

The optimal excitation and emission wavelengths of free fullerene were determined through spectrofluorometry, employing a broad range of measurements, using spectrofluorometry (BMG Labtech, Germany). Then, the release rate of fullerene from the fullerene@CA nanofiber scaffold was assessed after 6, 24, 48, and 72 h incubations in phenol red free-culture medium via measuring it at excitation of 355 nm and emission of 590 nm.

### Determination of cytotoxicity of fullerene@CA nanocomposite fibers to normal human cell line

Normal human lung fibroblast Wi-38 cell line (passage#30) was used to detect cytotoxicity of the prepared Fullerene@CA nanocomposite fibers, supplied from the American type culture collection (ATCC, USA). Wi-38 cell line was cultured in DMEM medium 10% fetal bovine serum (FBS), seeded as 5 × 10^3^ cells per well in a 96-well cell culture plate, and incubated at 37 °C in a 5% CO_2_ incubator. After 24 h for cell attachment, 0.1, 0.3 and 0.5 mg of free-fullerene and Fullerene@CA nanocomposite fibers were incubated with Wi-38 cells for 72 h. Cell viability was assayed by the MTT method. The wells were filled with 20 μl with 5 mg/ml MTT (Sigma, USA), and the plate was then incubated at 37 °C for 3 h. 100 μl of DMSO was added after the MTT solution was eliminated, and a microplate reader was used to measure each well's absorbance at 570 nm (BMG LabTech, Germany). The Graphpad Instat program calculated the investigated substances' effective, safe concentration (EC100) value (at 100% cell viability).

### Investigation of the anticancer activity

The anticancer effect of Fullerene@CA nanocomposite fibers was assayed using three human cancer cell lines that were obtained from (ATCC, USA). Colon cancer cell line (Caco-2, passage#32), triple-negative breast cancer cell line (MDA-MB 231, passage#35), and liver cancer cell line (HepG-2, passage#40) were cultured in DMEM (Lonza, USA) contained with 10% FBS (GEBCO, USA) supplemented with 10% FBS. All cancer cells (4 × 10^3^ cells/well) were seeded in sterile 96-well plates. After 24 h, 0.1, 0.3, and 0.5 mg.g^−1^ Fullerene@CA and CA free-fullerene were incubated with three cancer cell lines for 72 h at 37 °C in a 5% CO_2_ incubator. The MTT method was done as described above. The percentage of growth inhibition of three tested cancer cell lines was calculated at each corresponding dose, relative to the untreated cells. Furthermore, cellular morphological changes before and after treatment with the most effective and safest anticancer compounds were investigated using a phase contrast inverted microscope with a digital camera (Olympus, Japan).

### Flow cytometric analysis of the apoptotic effect of the tested anticancer compounds

The tested samples CA, Fullerene@CA nanocomposite fibers were incubated, for 72 h, with a Caco-2 cell line. Both untreated and treated cells were trypsinized before being incubated with annexin V/PI for 15 min. Quantification of annexin-stained apoptotic cells utilizing the FITC signal detector (FL1) versus the phycoerythrin emission signal detector was used to investigate the apoptosis-dependent anticancer impact (FL2).

### Quantitative detection for the change in the expression of proapoptotic genes and oncogenes in the treated cancer cells

The Gene JET RNA Purification Kit recovered total RNAs from Caco-2 cells treated with the studied anti-cancer drugs and left untreated (Thermo Scientific, USA). The mRNA was converted into cDNA (Thermo Scientific, USA) using a cDNA Synthesis Kit. SYBR green master mix was used for real-time PCR. The used primers (Forward/Reverse) were 5′-CTGGTGGACAACATCGCCCT-3′/5′-TCTTCAGAGACAGCCAGGAGAAAT-3′, 5′-TACTCTGGCGCAGAAATTAGGTC-3′/5′-CTGTCTCGGAGCTCGTCTATTTG-3′, 5′-TAACAGTTCCTGCATGGGCGGC-3′/5′-AGGACAGGCACAAACACGCACC-3′, 5′-CCGCCGTGGACACAGAC-3′/5′-CAGAAAACATGTCAGCTGCCA-3′, and 5′-CTGGGGATGTCCGTCAGAAC-3′/5′-GCCATTAGCGCATCACAGT-3′ for BCL2, NF-KB, and p53, Bax and p21 genes, respectively. B-actin (F: AAGCAGGAGTATGACGAGTCCG; R: GCCTTCATACATCTCAAGTTGG). The 2^−ΔΔCT^ equation was used to estimate the change in gene expressions in the treated cancer cells relative to untreated cancer cells using housekeeping gene B-actin.

### Statistical analysis

The data are expressed as mean ± standard error of the mean (SEM) and the significant values were considered at *p* < 0.05. One-way analysis of variance (ANOVA) by Tukey’s test was used to evaluate the difference between the mean values of the studied treatments. The analysis was done for three measurements using SPSS software version 16.

## Results and discussion

### Characterization of fullerene@CA nanocomposite fibers

**Figure 1:**
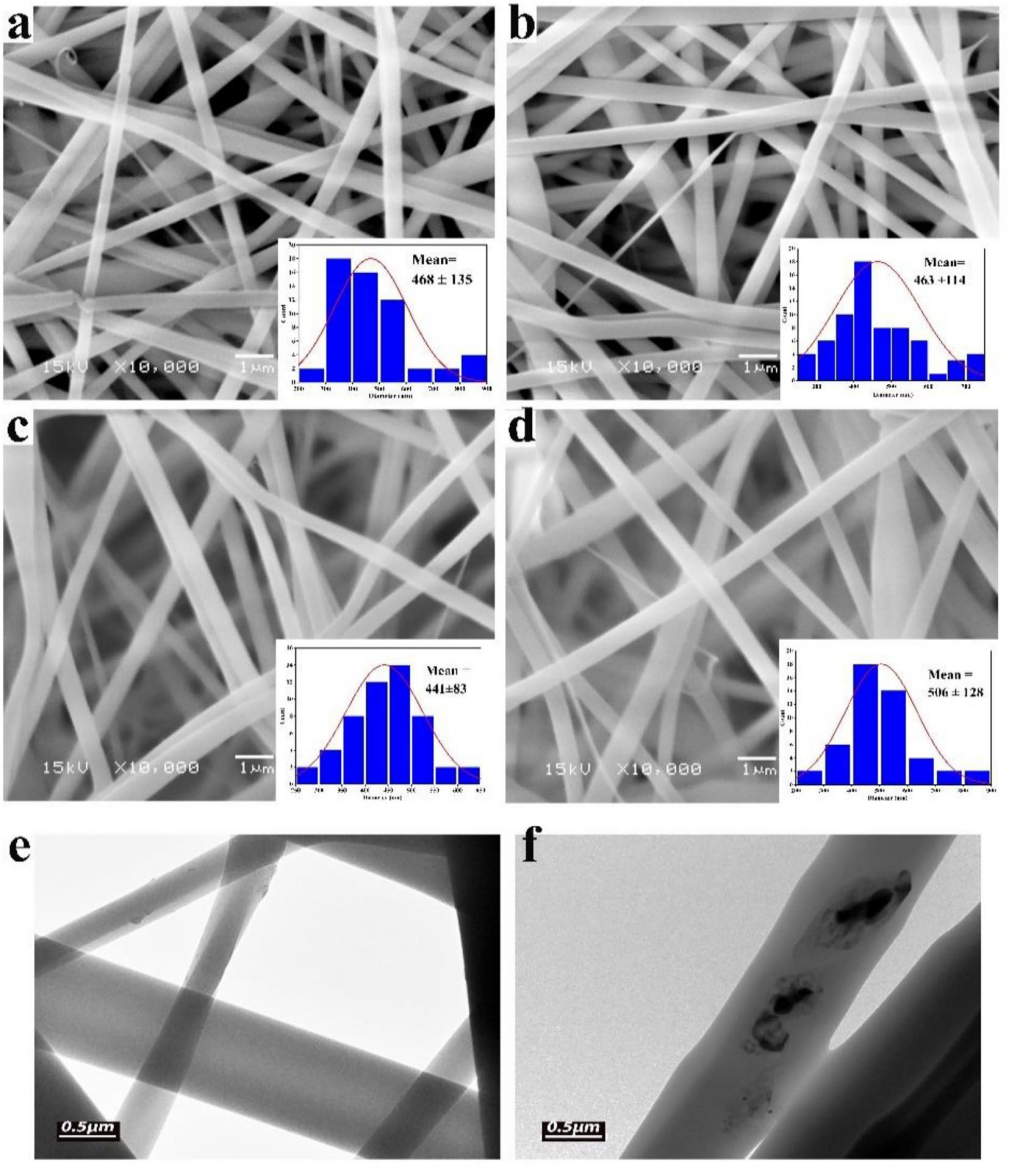
SEM images and diameter distribution histograms of CA and Fullerene@CA nanocomposite fibers loaded with different concentrations of fullerene: (**a**) without adding fullerene, and with (**b**) 0.1 mg.g^−1^, (**c**) 0.3 mg.g^−1^, and (**d**) 0.5 mg.g^−1^; and their diameter distribution; TEM images of (**e**) CA and (f) Fullerene@CA.

SEM images for CA and Fullerene@CA nanocomposite fiber are shown in Fig. 1(a,b,c and d). Evidently, smooth, bead-free nanofibers are obtained and no fullerene aggregates are observed on the surface of these nanofibers, implying that the as-loaded fullerene is perfectly incorporated well within the nanofibers. The diameter of the neat CA nanofibers are 486 nm. These results align with the previous work of Suwantong et al. when CA was used for biomedical applications and the average diameter was 300 nm^45^. However, the diameter in our work is higher than that due to the solvent effect we used. Solvent has a critical role in determining the shape and diameter of the nanofiber^26^. In contrast, the diameters of the Fullerene@CA nanofiber ranged between 441 and 506 nm with no particular dependency on the initial amount of the as-loaded fullerene.

TEM analysis was conducted to observe fluorine particles that were loaded into the CA nanofibers (Fig. 1e and f). The pristine CA nanofiber is homogenous and does not possess any particles derived from fluorine (Fig. 1e). The image of Fullerene@CA (Fig. 1f) reveals the presence of fluorine-associated particles with varying sizes. The aforementioned findings are consistent with earlier TEM analysis of CA when incorporated with TiO_2_ and AgNPs and also indicate a similar outcome^46^.

**Figure 2:**
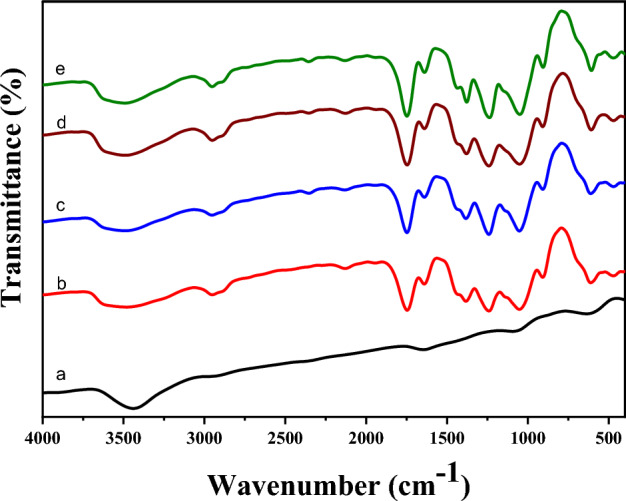
FTIR spectra of (**a**) fullerene and Fullerene@CA nanocomposite fibers: (**b**) without adding fullerene, and with (**c**) 0.1 mg.g^−1^, (**d**) 0.3 mg.g^−1^, and (**e**) 0.5 mg.g^−1^.

FTIR spectra for CA and Fullerene@CA nanocomposite fibers with different loading ratios are presented in Fig. 2. Prepared fullerene has a band around 1650 cm^−1^ corresponding to stretching carbonyl group (C = O) and C = C vibration, in addition to the band at 1095 cm^−1^ which was ascribed to the intrinsic characteristic of sp2 graphitic materials and oxygen functionalities of skeletal stretching vibration of C = O group and the broad peak at 3441 cm^−1^ representing O–H stretching vibration of hydroxyl functional groups (Fig. 2a)^43,44^. CA has strong peaks at 3455 cm^−1^ and 1044 cm^−1^ which are assigned to the hydroxyl group and ring of cellulose ether (Fig. 2b)^47^. Furthermore, it has characteristic peaks of C = O, C-O, and C-H of the acetyl group (CHCOO) at 1755 cm^−1^, 1384 cm^−1^, and 1234 cm^−1^, respectively^48^. These results agree with earlier FTIR analyses for CA^49^. Fullerene@CA nanocomposite fibers show the same peaks for CA and fullerene. Nevertheless, the peaks have more intensity and become sharper (Fig. 2 c,d,e). FTIR data indicate that the prepared fullerene has been successfully incorporated into CA fibers.

**Figure 3:**
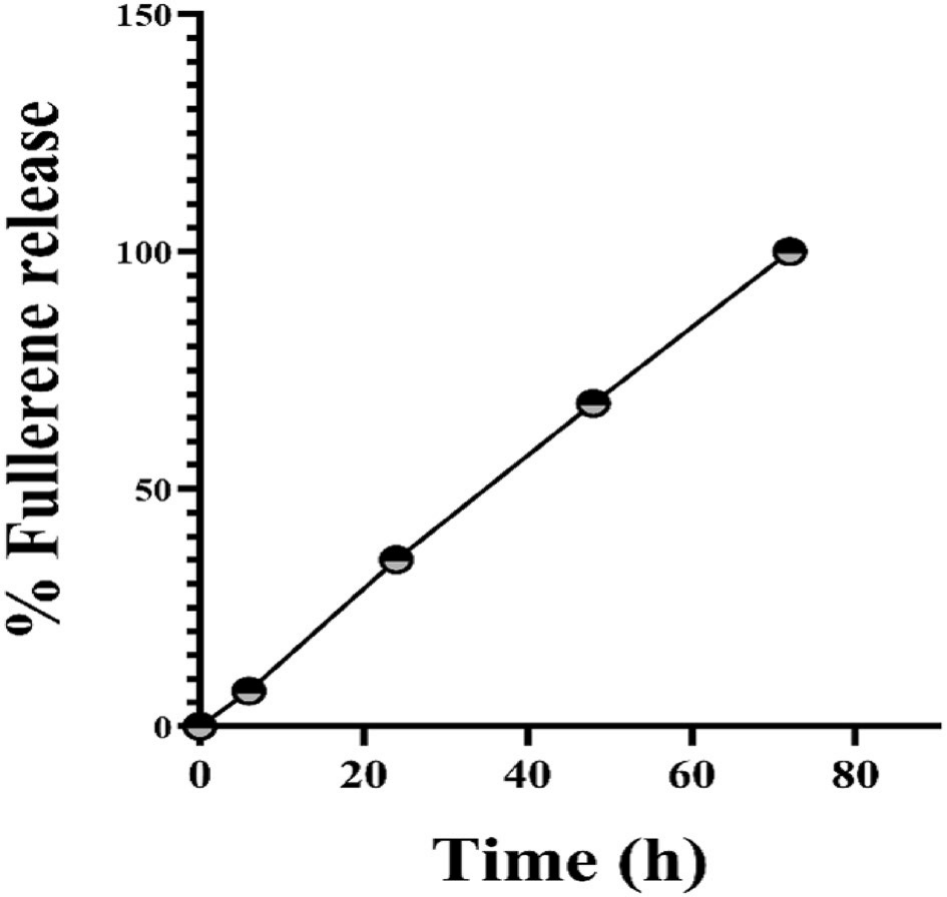
Release profile of Fullerene from nanofiber at 6, 24, 48, and 72 h.

As demonstrated in Fig. 3, the release rate of fullerene increased in a time-dependent manner from 7.43, 34.96, and 67.69% to 99.46% at 6 h, 24 h, and 48 h, respectively, to 72 h.

### Cytotoxicity of fullerene@CA nanocomposite fibers to normal human cell line

An assessment of their safety profile was carried out using cytotoxicity screening on normal fibroblasts (Wi-38), followed by a comparison of their anticancer potential with 5-fluorouracil (5-FU) against three selected cancer cell lines (Caco-2, MDA-MB 231 and HepG-2). Fullerene@CA nanocomposite fibers, loaded with fullerene concentrations (0.1, 0.3, and 0.5 mg.g^−1^), are initially used as prescription carriers for anticancer treatments because fullerene has anticancer properties—according to previous studies^50–52^. The effect of Fullerene@CA nanocomposite fiber as an anti-cancer material was revealed, and it began to be evaluated as a standalone drug for anti-tumor growth where Bacakova et.al. investigated nanocellulose/nanocarbon composites in biomedical applications^53^. In contrast, Hamouda et al. use Au/cellulose nanocomposite as an anticancer^54^. However, numerous markers are used in cell viability studies to identify metabolically active (living) cells^55^.

**Figure 4:**
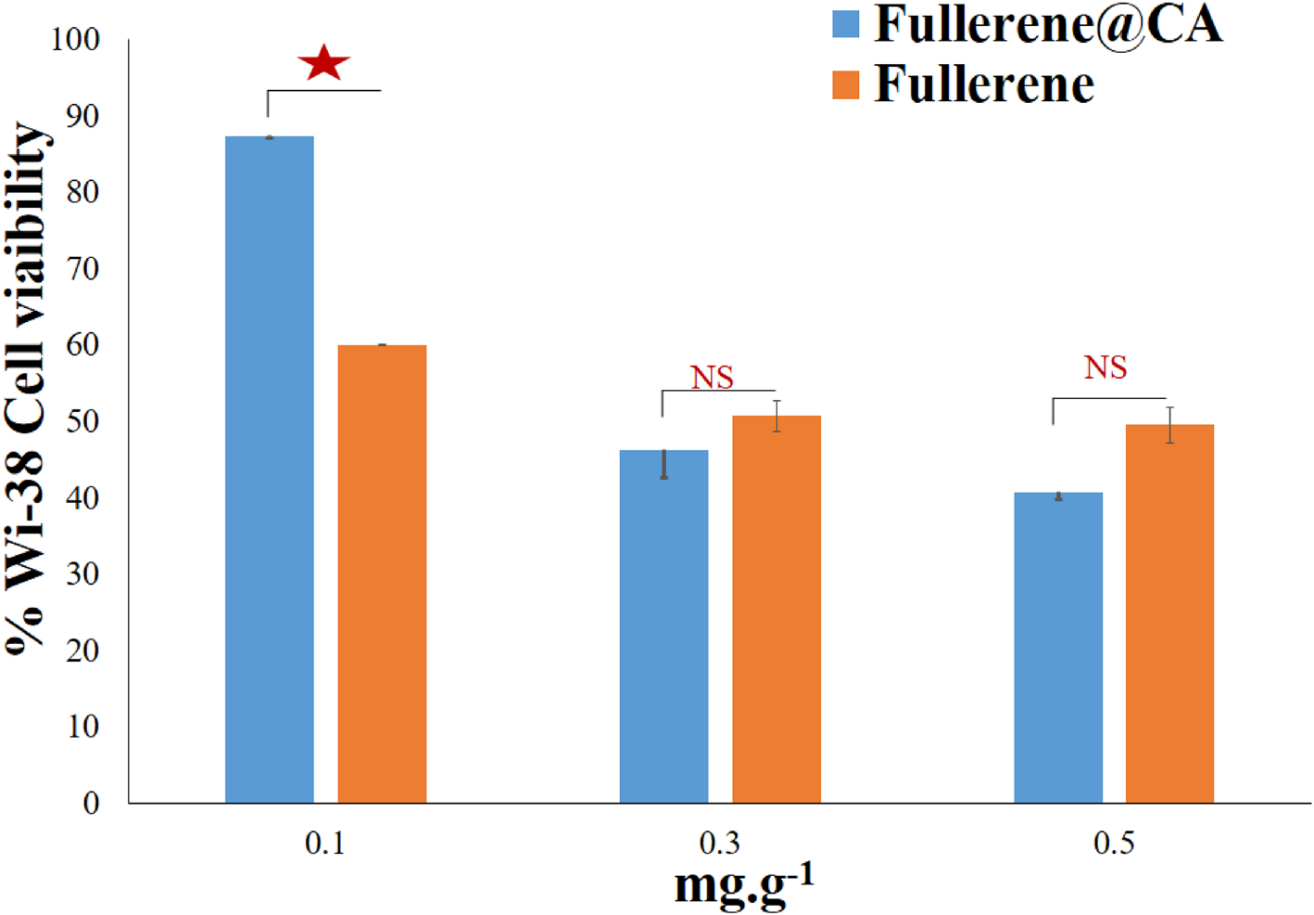
The cytotoxicity effect of Fullerene@CA nanocomposite fibers with different fullerene-loaded ratios on Wi-38 cell viability compared to free-fullerene. (Value are presented as mean ± SEM and considered statistically significant at * *P* ≤ 0.05. NS refers to non-significant variation).

The Fullerene@CA nanocomposite fibers loaded with 0.1 mg fullerene-maintained Wi-38 cell viability above 87% relative to < 60% in the case of fullerene which stands alone as free-fullerene. Conversely, when considering higher concentrations (0.3 and 0.5 mg), there are no significant differences in the viability of regular cells (< 51%) when comparing the free-fullerene and Fullerene@CA forms (Fig. 4).

### Investigation of the anticancer activity

**Figure 5:**
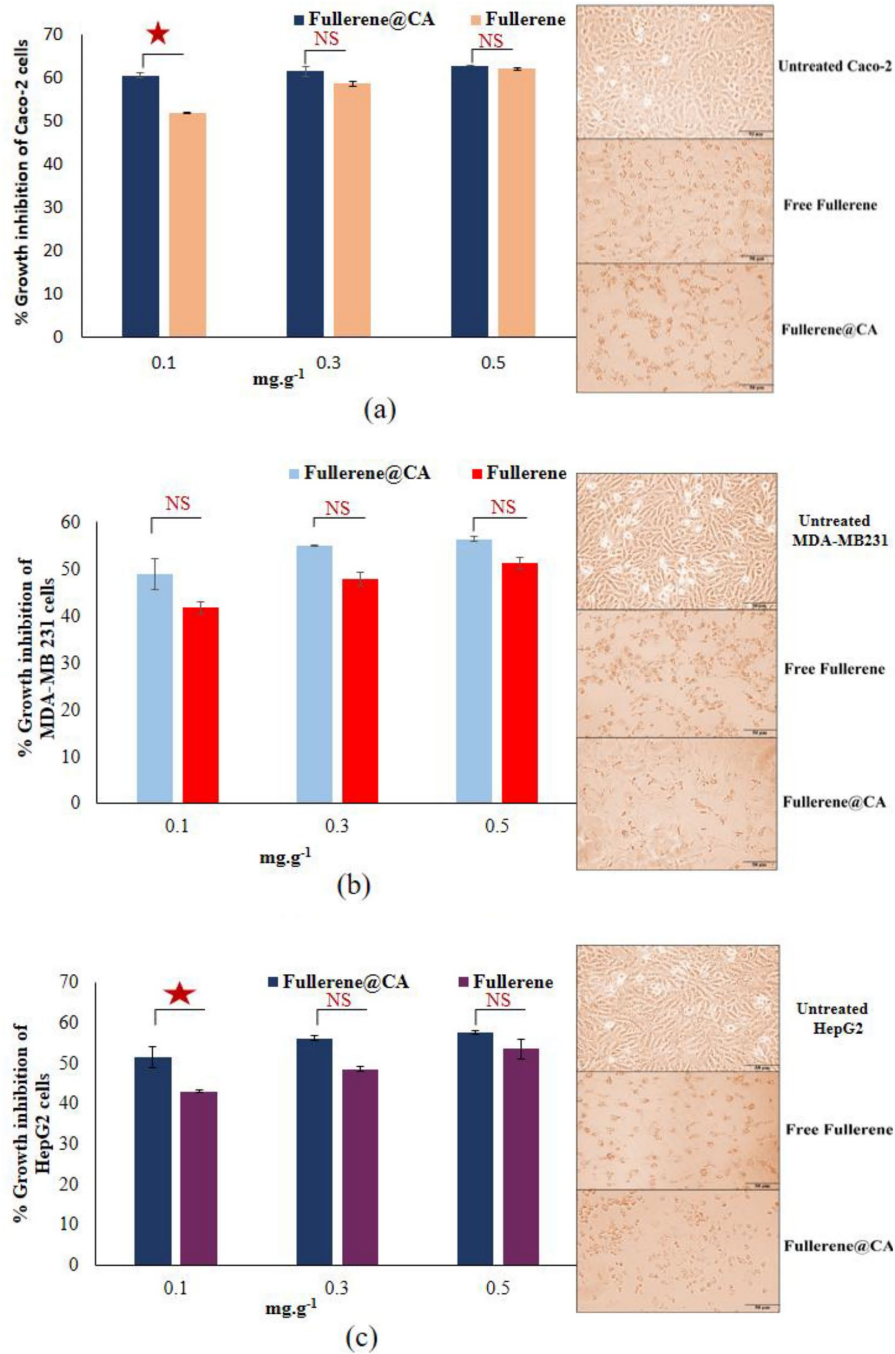
The cytotoxicity effect of fullerene and Fullerene@CA nanocomposite fibers with different fullerene loaded ratios on the percentage of growth inhibition after 72 h incubation and morphological changes of the treated (**a**) Caco-2, (**b**) MDA-MB 231 and (**c**) HepG2 cells with 0.1 mg of fullerene, as free and fiber loaded compound, compared to the untreated cancer cells. (Value are presented as mean ± SEM and considered statistically significant at * *P* ≤ 0.05. NS refers to non-significant variation), (Magnification 100 X; scale bar 50 µm).

Included: cytotoxicity of the tested compounds on human cancers cell lines, flow cytometric analysis of the apoptotic effect of the tested compounds, and relative change in the expression of critical genes in the treated cancer cells. CA fibers and Fullerene@CA nanocomposite fibers exhibited potent anticancer activity, in a dose-dependent manner, against Caco-2, MDA-MB 231, and HepG-2 cells. Figure 5 demonstrates that Fullerene@CA nanocomposite fibers loaded with 0.1, 0.3, and 0.5 mg.g^−1^ of fullerene inhibited the growth of these cancer cells by 58.62–62.87, 47.86–56.43%, and 48.60–57.73%, respectively. However, the lowest dose of the loaded fullerene (0.1 mg.g^−1^) significantly suppressed the proliferation of the studied cancer cell lines by 60.56 ± 0.56, 49.00 ± 3.29 and 51.53 ± 2.47%, for Caco-2, MDA-MB 231 and HepG-2 cells respectively, compared with free-fullerene that stands only form 51.94 ± 0.06, 41.71 ± 1.29 and 43.07 ± 0.40%, respectively. In addition, it has been observed that at this particular concentration, the safest dosage tested on Wi-38, there are morphological changes in human cancer cells, such as cell shrinkage and the loss of spindle shape. The CA fibers and Fullerene@CA nanocomposite fibers exhibit distinct variations in their efficient experiences with respect to size reduction. This finding is primarily attributed to the confinement of electrons to smaller surfaces and the corresponding increase in the edge-plane ratio. The increased density of edges facilitates a greater degree of electrochemical activity. Due to the higher edge density, this enables more electrochemical activity. Despite being less electrochemically active, the oxygenated equivalents exhibit superior stability and dispersibility, rendering them highly effective in cancer treatment^56^. Conversely, fullerenes have received much attention due to their potential use in human medicine^57^.

Several studies have employed cellulose nanofibers in the treatment of cancer cells. For instance, the combination of cellulose nanofibers with a bilayer of poly(diallyldimethylammonium chloride) (PDADMAC) and polyacrylic acid (PAA) through electrostatic interactions has demonstrated promising outcomes. This can be attributed to the negative surface charge exhibited by cellulose fibers^58^. In addition, gold-silver NPs, electrospun silk fibroin, and CA (CA/SF/Au–Ag) composite nanofiber were used as anticancer where the fabricated CA/SF/Au–Ag nanofiber has an efficient IC50 value and substantially induced the cytotoxic effects against the human breast cancer cells MCF-7 and MDA-MB-231^59^. A critical step in drug screening and cancer research is the development of nanofibrous scaffolds for in vitro cancer cells using graphene oxide (GO) and (CA)^60^. The mechanical properties of GO/CA scaffolds exhibited a clear superiority over bare CA scaffolds, with an observed enhancement as the concentration of GO increased. In vitro cell experiments revealed that cancer cells exhibited significantly enhanced vitality, improved cell adherence, and accelerated growth when cultured on GO/CA scaffolds compared to those cultured on bare CA scaffolds. To the best of our understanding, there has been no prior investigation into the utilization of nanocomposite electrospun fibers comprising fullerene NPs with cellulose acetate (CA) for the specific targeting and treatment of various cancer cell types, including colon cancer cell line (Caco-2), breast cancer cell line (MDA-MB 231), and liver cancer cell line (HepG-2). Therefore, this study contributes a novel discovery to the existing body of knowledge in this particular domain.

### Flow cytometric analysis of the apoptotic effect of the tested anticancer compounds

**Figure 6:**
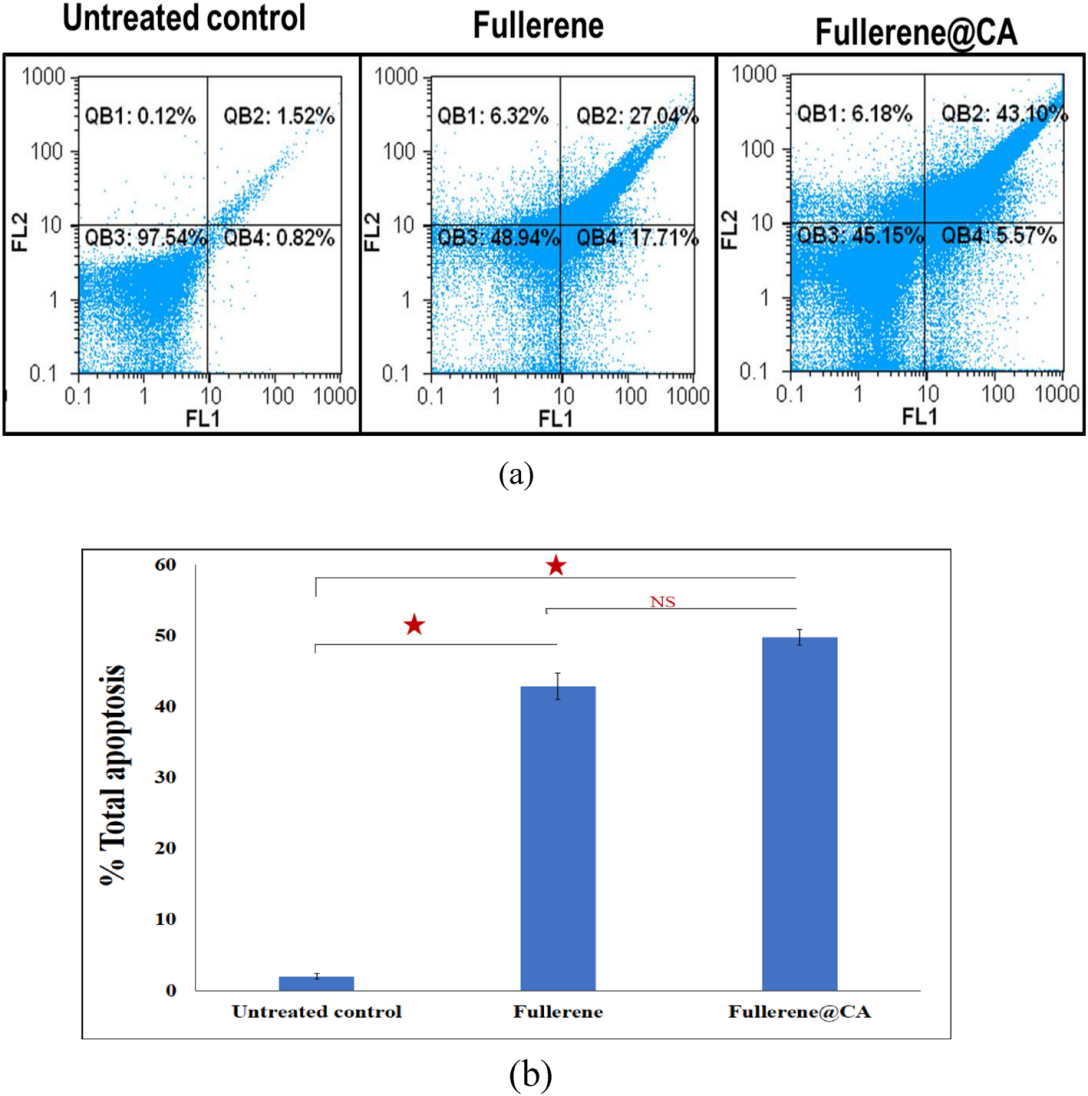
Flow cytometric analysis of Fullerene@CA nanocomposite fibers loaded with 0.1 mg.g^−1^ treated Caco-2 cells, after 72 h incubation, comparing with the untreated control cells and treated cell with fullerene only, (**a**) Dot charts of Annexin-propidium iodide-stained cells (FL1/FL2) with (**b**) the apoptosis percentages. (Value are presented as mean ± SEM and considered statistically significant at * *P* ≤ 0.05. NS refers to non-significant variation).

Quantification of annexin-stained apoptotic cells was done and the apoptotic powerful effect on Caco-2 was confirmed by a high percentage of annexin-stained apoptotic population after 72 h treatment relative to the untreated cells (Fig. 6 a,b). The Fullerene@CA nanocomposite fibers loaded with 0.1 mg.g^−1^ have more substantial apoptotic potential than free-fullerene, where the loaded fiber can induce apoptosis by 49.79 ± 1.12% compared to 42.88 ± 1.86% in fullerene-treated Caco-2. The production of ROS in cells clarified the molecular pathways behind fullerene-induced apoptosis^61^. Fullerene improved the ROS-independent MEK-ERK pathway stimulation composed of the extracellular signal-regulated kinase (ERK) and mitogen-activated protein kinase (MAPK)^62^.

### Quantitative detection for the change in the expression of treated (Caco-2, MDA-MB 231, and HepG-2 cells)

**Figure 7:**
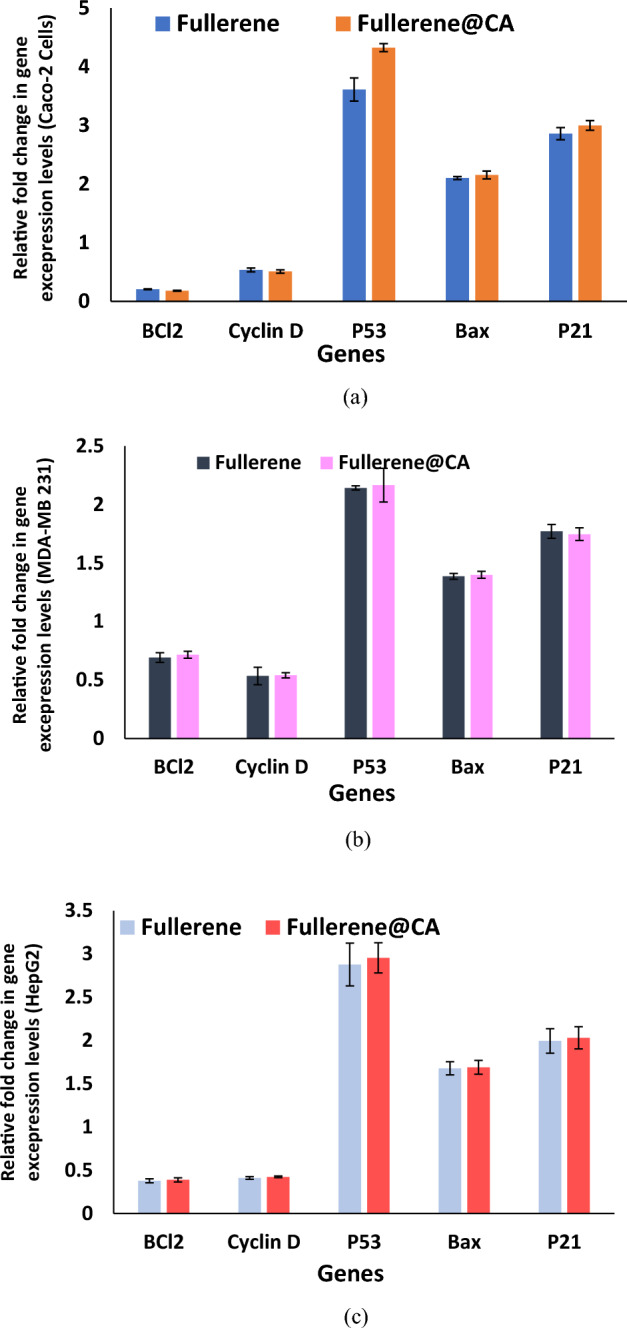
Relative fold change in the expression of oncogenes and apoptotic genes in loaded CA fibers and Fullerene@CA nanocomposite fibers loaded with 0.1 mg.g^−1^-treated (**a**) Caco-2, (**b**) MDA-MB 231 and (**c**) HepG-2 cells. (Value are presented as mean ± SEM).

qRT-PCR analysis of genes expression RT-PCR analysis of cells after treatment with fullerene and Fullerene@CA nanocomposite fibers was studied to determine the differences after treatment of cancer cells as presented in other studies. Both materials are nearly equipotent in the treated Caco-2 cells, critical genes of proliferation and apoptosis are measured at the mRNA level. Both forms can suppress the expression of BCl2 and cyclin D by ~ 5 and 2 folds, respectively. Furthermore, in its loaded and free forms, fullerene can induce the expression of p53 by 4 and 3 folds, respectively. The downstream (Bax and p21 genes) are also upregulated by 2 and 3 folds, respectively, in the treated cells, with no significant difference between loaded and free (Fig. 7).

Multiple genes play a crucial role in the regulation of cellular division and developmental processes. The cell cycle refers to the systematic and sequential mechanism through which a cell undergoes replication to produce an identical copy of itself. The aforementioned process is subject to stringent regulation in order to ensure the accurate duplication of DNA in dividing cells, the rectification of any DNA errors, and the equitable distribution of a complete set of chromosomes to each progeny cell. The observed fold change in gene expression levels of the P53 gene, a known tumor suppressor gene, is higher in Caco-2 cells than other genes utilized to demonstrate the genetic impact on treated Caco-2 cells. In contrast, utilizing Fullerene@CA nanocomposite fibers with a loading concentration of 0.1 mg.g-1 demonstrates greater efficacy than pure fullerene. Specifically, the relative fold change in gene expression levels of p53 increased by 0.72 in the presence of Fullerene@CA nanocomposite fibers. Following the occurrence of double-stranded DNA strand breaks, the levels of p53 increase due to the stabilization process. Additionally, kinases promptly phosphorylate histone H2AX at or in proximity to the sites of DNA damage, resulting in the formation of C-H2AX^63,64^. The p53 protein in the cell binds DNA, activating a different gene to make the p21 protein, which interacts with a protein that promotes cell division (cdk2). The relative fold change in gene expression levels of the BAX gene for Caco-2 cell increases than NF-KB and less than (P53 & p21) where, the first pro-apoptotic member of the Bcl-2 protein family to be discovered is the BAX gene^65^. A moderating variable's value affects how the mediating variable (lincRNA-p21) behaves^66^. Based on the current data, lincRNA-p21 is a promising indicator of hematotoxicity therapy response. While, BCL-2, an anti-apoptotic protein, is found to have significantly decreased levels; this links the research materials to the apoptotic process in Caco-2 cells. The interaction between BCL-2 and VEGFA (vascular endothelial growth factor A) has been investigated in several cancer cells. BCL-2 has been shown to increase the expression of the VEGFA gene^67,68^.

## Conclusion

This study presents a comprehensive methodology for the fabrication of biocompatible electrospun cellulose acetate (CA) materials specifically designed for use in biomedical applications. The incorporation of fullerene, a carbonaceous nanomaterial, was carried out in CA nanofibers at varying weight ratios (0.1, 0.3 and 0.5 mg.g^−1^). The addition of fullerene to CA nanofiber showed an insignificant effect on the morphology of neat CA. FTIR data confirmed the presence of fullerene in CA nanofiber (Fullerene @CA). The cytotoxicity of CA free-fullerene and Fullerene@CA nanocomposite fiber was assessed against Wi-38 cells. It was observed that the fullerene encapsulation (0.1-Fullerene@CA) demonstrated the ability to protect normal cells while still exhibiting its anticancer properties against Caco-2, MDA-MB 231, and HepG-2 cells. The findings indicate that Fullerene@CA nanocomposite fiber prepared by electrospinning could be a promising candidate for biomedical applications as a cancer-fighting material. Based on the current study data, further research is required to validate the findings obtained in this area.

## Data Availability

All data generated or analyzed during this study are included in this published article.
